# Artificial intelligence–enabled electrocardiography for assessment of left ventricular systolic dysfunction in the era of foundation models

**DOI:** 10.3389/fcvm.2026.1899224

**Published:** 2026-08-24

**Authors:** Andreas Bollmann, Victoria Pradler, Daniela Husser, Igor Kim, Sven Hohenstein, Anne Nitsche, Stefan Kwast, Olaf Kannt, Christian Pawlu, Alexander Moscho, Ralf Kuhlen

**Affiliations:** 1 Helios Health Institute, Leipzig, Germany; 2 Fresenius, Bad Homburg, Germany; 3 Medical Faculty, University of Muenster, Muenster, Germany; 4 Department of Electrophysiology, Heart Center Leipzig at University of Leipzig, Leipzig, Germany; 5 Helios Hospitals, Berlin, Germany

**Keywords:** artificial intelligence, electrocardiography, foundation models, heart failure, left ventricular ejection fraction, screening

## Abstract

Artificial intelligence (AI) applied to the standard 12-lead electrocardiogram (AI-ECG) is being developed as a scalable approach to screen for left ventricular systolic dysfunction (LVSD) and support triage for confirmatory testing. Supervised models trained on paired ECG–echocardiography data show high discrimination for reduced ejection fraction across thresholds and can identify individuals at higher risk of subsequent LV dysfunction despite a normal baseline echocardiogram. External validation of an FDA-cleared ECG-AI device across four geographically diverse U.S. health systems confirmed strong diagnostic accuracy, though signal-format compatibility and quality gating meaningfully affect real-world yield. Two pragmatic randomized trials demonstrate practice-level impact. In primary care, AI-ECG increased the number of new low-ejection-fraction diagnoses and directed echocardiography preferentially to screen-positive patients. In non-cardiology inpatient wards, AI alerts improved diagnostic yield through increased cardiology consultation rather than increased imaging volume. In emergency-department patients with dyspnea, AI-ECG supports a prioritization role with high negative predictive value, outperforming NT-proBNP, but requires confirmatory imaging given prevalence-dependent positive predictive value. In population cohorts, adding AI-ECG signals to PREVENT-HF improves near-term heart-failure risk discrimination and reclassification, though without demonstrated benefit on clinical outcomes such as heart-failure hospitalization or mortality. Foundation models pretrained on large ECG datasets reduce labeled-data requirements and improve transportability, but prospective echocardiography-anchored validation is required before broader deployment. FDA-cleared software is available for left ventricular ejection fraction ≤40% screening from 12-lead ECGs as clinician decision support. This review summarizes performance across thresholds and care settings, outlines threshold selection and calibration, and defines priorities for outcome-oriented trials, equitable deployment, and implementation governance.

## Introduction

Heart failure (HF) affects more than 64 million people globally, with prevalence rising as populations age and survival after myocardial infarction improves (1). Earlier identification of left ventricular systolic dysfunction (LVSD) enables timely initiation of guideline-directed therapies and may reduce downstream events. Since 2019, artificial-intelligence–enabled electrocardiography (AI-ECG) is being developed as a scalable approach to screen for reduced left ventricular ejection fraction (LVEF) from 10-s, 12-lead ECG waveforms. Multiple studies have reported high discrimination for prevalent LVSD and have linked AI-ECG outputs to future development of LV dysfunction, suggesting that ECG waveforms contain informative signatures beyond conventional interpretation (2, 3). Recent retrospective analyses in pooled community cohorts further suggest that combining AI-ECG signals (including screens for systolic and diastolic dysfunction) with clinical risk equations (e.g., PREVENT-HF) can improve near-term risk discrimination and reclassification for incident HF within the broader PREVENT framework (4–6). In parallel, ECG foundation models pretrained on large ECG datasets may reduce labeled-data requirements and improve transportability when fine-tuned for LVSD tasks (7–10).

## Scope and methods

**Figure 1:**
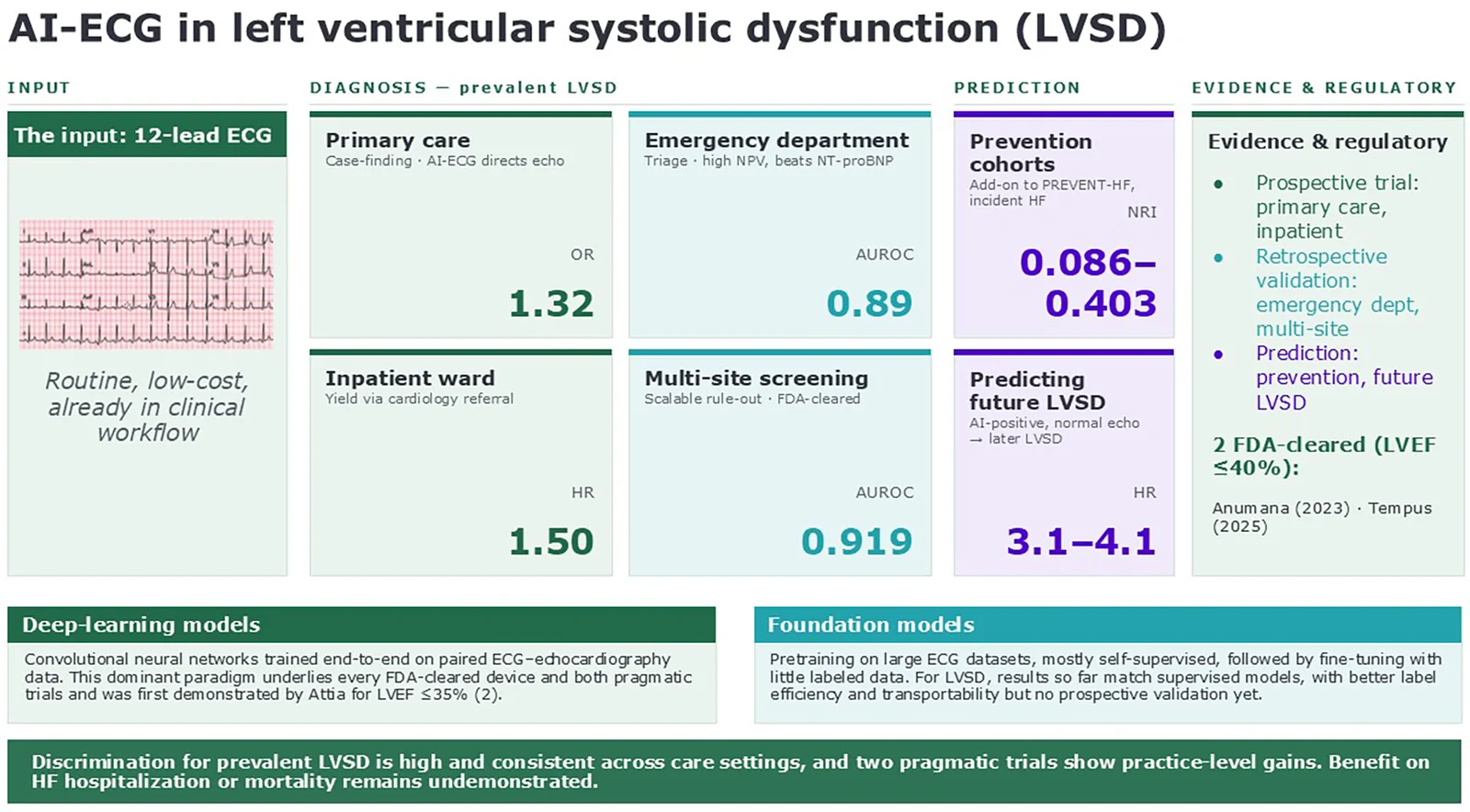
Overview of AI-ECG for left ventricular systolic dysfunction. The 12-lead ECG is used both to detect prevalent LVSD across care settings and to predict future LV dysfunction or incident heart failure, with models built either as supervised deep-learning systems or as self-supervised foundation models. Discrimination for prevalent LVSD is high and consistent across settings, FDA-cleared tools are available for clinical decision support, and benefit on heart-failure hospitalization or mortality is not yet established. Setting-specific estimates, study designs, and regulatory status are detailed in Table 1. AUROC, area under the receiver operating characteristic curve; ECG, electrocardiogram; FDA, U.S. Food and Drug Administration; LV, left ventricular; LVSD, left ventricular systolic dysfunction.

This review summarizes the diagnostic performance, external validation, and pragmatic implementation of AI-ECG for LVSD across primary care, inpatient, emergency-department, and prevention settings, and outlines practical considerations for thresholding, calibration, and outcome-focused evaluation. Two methodological classes are considered, supervised deep-learning models and foundation models, with evidence spanning randomized trials, external validation, prevention cohorts, and benchmark evaluations. MEDLINE/PubMed was searched through May 2026, and studies were selected to represent each methodological class across these care settings, with priority given to those reporting cohort-level performance against a clinical or imaging reference standard and to those providing external validation or prospective evidence. The review is narrative in scope and does not claim systematic-search reproducibility. The individual methods, cohorts, and databases are enumerated in Tables 1, 2, where each dataset is characterized as institutional or public and by its role in the cited study, and the overall framework is summarized in Figure 1. All performance and implementation recommendations refer to 10-s, 12-lead ECG inputs, the modality with prospective validation for LVSD detection.

**Table 1:** AI-enabled ECG for LVSD screening and risk stratification: performance across care settings and model types.

Care setting	LVEF threshold	Model type	Study design	*n*	Key performance	Comparator	Clinical role	Key caveat
Primary care (18)	≤50%	Supervised deep learning	Cluster RCT	22,641	↑ new low-EF diagnoses (OR 1.32); ↑ diagnosis in AI-positive subgroup (OR 1.43); overall echo utilization unchanged	Standard care (no AI-ECG result displayed)	Case-finding; 2-stage AI-ECG → echo workflow	No HF hospitalization or mortality endpoint
Inpatient ward (non-cardiology) (19)	≤50%	Supervised deep learning	Pragmatic RCT	13,631	↑ new low-EF diagnoses (HR 1.50); improved echocardiography yield in AI-positive patients (PPV 34.2% vs. 20.2%); overall echo utilization unchanged	Standard care (no AI-ECG alert)	Case-finding; improved diagnostic yield via cardiology consultation pathway	Single center (Taiwan); no mortality benefit demonstrated
Emergency department (dyspnea) (20)	≤35%	Supervised deep learning	Retrospective, single-center	1,606	AUROC 0.89; sensitivity 74%; specificity 87%; NPV ∼97%; PPV ∼40%	NT-proBNP >800 pg/mL (AUROC 0.80)	Triage/prioritization; de-prioritize echo in low-risk; not stand-alone rule-out	Single center; PPV prevalence-dependent; confirmatory echo required
Multi-site external validation (general/screening) (12)	≤40%	Supervised deep learning (FDA-cleared SaMD)	Retrospective, 4-site external validation	13,960	AUROC 0.919; sensitivity 84.5%; specificity 83.6%; NPV 98.4%; PPV 30.5%; 78% test-negative rate	Pre-specified performance benchmarks (sensitivity/specificity each >80%)	Scalable screening; rule-out strategy to defer echo in low-risk patients	2,040/16,000 ECGs nonconformant; paced rhythms excluded; intention-to-diagnose sensitivity 76.5%
Prevention/community cohorts (4)	incident HF risk	Supervised deep learning	Retrospective pooled cohort	14,126	NRI 0.086–0.125 at PREVENT-HF ≥10%; NRI 0.327–0.403 at PREVENT-HF ≥20%	PREVENT-HF equation alone	Add-on risk stratification for incident HF across 1–10 years	Retrospective; no clinical outcome benefit demonstrated
Cross-setting (foundation models—scale) (7, 9)	Multiple (LVEF classification/regression)	Foundation model, supervised multilabel pretraining (ECGFounder) Self-supervised transformer (Moody et al.)	Retrospective benchmark/multi-cohort evaluation	ECGFounder: >10M pretraining; Moody et al.: 800,035 pretraining; fine-tuning on task-specific cohorts	ECGFounder: cross-domain generalization across 150 label categories; LVEF transfer demonstrated. Moody et al.: AUROC 0.955 overall and 0.949 external (0.949 SSL vs. 0.894 no-SSL) for LVEF <35%; SSL improved performance in 11/12 tasks vs. *de novo* supervised training	Task-specific supervised models trained from scratch	Label-efficient local adaptation; improved transportability across datasets and tasks	No prospective echo-anchored validation for LVSD screening; LVEF threshold <35% (Moody) differs from ≤40% standard; ECGFounder additionally supports reduced-lead and single-lead input
Cross-setting (foundation models—LVSD-specific head-to-head) (8, 10)	≤40%	Foundation model self-supervised (DeepECG-SSL) vs. supervised (DeepECG-SL) Open-weight ECG-FM	Retrospective benchmark, external validation	Nolin-Lapalme et al.: >1M pretraining ECGs; validated across 11 datasets (881,403 ECGs). ECG-FM 1.5M pretraining ECGs; UHN-ECG for LVEF	DeepECG-SSL AUROC 0.926 vs. DeepECG-SL 0.917 for LVEF ≤40% (Δ = 0.009, *p* < 0.001); SSL advantage widens with decreasing training data. ECG-FM: AUROC 0.929 for LVEF ≤40%; strong label efficiency and cross-dataset generalization	Task-specific supervised models trained from scratch	Label-efficient local adaptation; SSL advantage most pronounced in data-limited settings	Pretraining and fine-tuning datasets partially overlapping in some implementations; no prospective LVSD screening validation; 12-lead input only

AUROC, area under the receiver operating characteristic curve; CHS, Cardiovascular Health Study; ECG, electrocardiogram; FHS, Framingham Heart Study; HR, hazard ratio; LVEF, left ventricular ejection fraction; LVSD, left ventricular systolic dysfunction; MESA, Multi-Ethnic Study of Atherosclerosis; NPV, negative predictive value; NRI, net reclassification improvement; PPV, positive predictive value; PREVENT-HF, Predicting Risk of Cardiovascular Disease EVENTs–Heart Failure; RCT, randomized controlled trial; SaMD, software as a medical device; SSL, self-supervised learning.

An independent head-to-head comparison of 4 of 51 published supervised AI-ECG models using paired ECG and cardiac magnetic resonance imaging as reference standard (*n* = 1,203) reported AUROCs 0.83–0.93; performance was attenuated with atrial fibrillation and wide QRS; only 4 of 51 identified models were available for independent testing (13).

**Table 2:** Methods and data inventory. Datasets are characterized by type (institutional versus public) and by role.

Study (ref)	Institutional dataset(s) (role)	Public dataset(s) (role)	Study purpose
Attia et al. (2)	Mayo paired ECG–TTE (training; internal validation)	–	Original development of the AI-ECG low-LVEF screen; flagged future-LVSD risk despite normal echo
Yao et al. (18)	Mayo primary-care cohort (deployment)	–	Pragmatic effectiveness in primary care
Tsai et al. (19)	Single-center inpatient cohort, Taiwan (deployment)	–	Pragmatic effectiveness and inpatient prognostication
Adedinsewo et al. (20)	Mayo emergency-department dyspnea cohort (retrospective application)	–	Retrospective application of an existing AI-ECG model to an ED dyspnea population
Carter et al. (12)	Four US health systems (external validation)	–	Multi-site external validation supporting FDA clearance
König et al. (24)	Heart Center Leipzig database and electronic medical records (external validation)	–	Independent European validation; future-LVSD prediction
Croon et al. (13)	Amsterdam UMC cardiac magnetic resonance registry (external validation)	–	First independent head-to-head comparison; reproducibility audit
Desai et al. (4)	–	FHS, MESA, CHS (pooled community cohorts)	Incremental value over PREVENT-HF for incident heart failure
Poterucha et al. (16)	NYP eight-hospital cohort (training; internal validation)	EchoNext (released benchmark)	Open paired-ECG benchmark; structural disease including low LVEF
Li et al. (7)	–	HEEDB (pretraining; internal validation); MIMIC-IV-ECG (fine-tuning); CODE-test, PTB-XL, PhysioNet 2017 (external validation)	General-purpose fine-tunable foundation model
McKeen et al. (8)	UHN-ECG (external validation)	PhysioNet 2021, MIMIC-IV-ECG (pretraining)	Open-weight foundation model; label efficiency
Moody et al. (9)	PET-labeled cohorts (fine-tuning; external validation); SPECT and CMR labels (external validation)	MIMIC-IV-ECG (pretraining); PTB-XL, UK Biobank (external validation)	Self-supervised foundation model for label-scarce functional tasks
Nolin-Lapalme et al. (10)	Montreal Heart Institute (training; internal validation); UCSF, UW, JGH, MGH, CSH, CHUM, NYP (external validation)	Code-15 (pretraining); MIMIC-IV-ECG (pretraining; external validation); CLSA, PTB-XL, UK Biobank (external validation)	Head-to-head supervised versus self-supervised; fairness and privacy

**Roles:** training (supervised development); pretraining (self-supervised); fine-tuning (downstream adaptation); internal validation; external validation; deployment (prospective or pragmatic clinical application of a fixed model).

**Inclusion** (Tables 1, 2)**:** these tables summarize primary studies that report cohort-level LVSD performance or provide independent external validation across the principal care settings and signal-based model classes covered by this review. Studies addressing specialized extensions, including single-lead acquisition, conduction-specific or arrhythmia subpopulations, metadata augmentation, image-based foundation models, and explainability-focused approaches, are discussed in the text.

CHS, Cardiovascular Health Study; CHUM, Centre Hospitalier de l'Université de Montréal; CLSA, Canadian Longitudinal Study on Aging; CMR, cardiac magnetic resonance imaging; CSH, Cedars-Sinai Hospital; FHS, Framingham Heart Study; HEEDB, Harvard-Emory ECG Database; JGH, Jewish General Hospital; MESA, Multi-Ethnic Study of Atherosclerosis; MGH, Massachusetts General Hospital; NYP, New York-Presbyterian; PET, positron emission tomography; SPECT, single-photon emission computed tomography; TTE, transthoracic echocardiography; UCSF, University of California San Francisco; UHN-ECG, University Health Network ECG; UW, University of Washington. Dataset names (MIMIC-IV-ECG, PTB-XL, Code-15, CODE-test, PhysioNet, UK Biobank, EchoNext) are used as published.

Public denotes datasets shared beyond the originating institution. Access tiers differ: PTB-XL, Code-15, and the PhysioNet challenge sets are open-access, whereas HEEDB, MIMIC-IV-ECG, UK Biobank, FHS, MESA, CHS, CLSA, and EchoNext are credentialed or controlled-access, requiring a data use agreement and, in some cases, ethics approval or training.

## First-generation supervised AI-ECG models

A supervised model by Attia et al. established both diagnostic performance for LVEF ≤35% and prospective risk stratification utility. In that study, individuals with an AI-positive ECG but a normal baseline echocardiogram were at higher risk of subsequently developing LVSD, supporting the hypothesis that AI-ECG may capture early electrical signatures that precede overt systolic dysfunction (2). Subsequent reviews report consistently high discrimination across cohorts and LVEF thresholds (≤35%, ≤40%, ≤50%), while also highlighting that much of the evidence base remains retrospective and sensitive to population mix and reference-standard timing (3, 11).

Recent work strengthens the external evidence base but also reveals persistent barriers to independent assessment. A multicenter U.S. external validation of a locked, FDA-cleared ECG-AI software device demonstrated strong diagnostic accuracy across geographically diverse health systems, though a substantial proportion of ECGs were nonconformant with device input requirements, underscoring the practical impact of signal-format compatibility and quality gating on real-world yield (12). An independent head-to-head comparison study found that true external validation was feasible for only 4 of 51 published LVSD AI-ECG models, with performance attenuated in the presence of atrial fibrillation and wide QRS, and substantial false-positive rates in borderline EF ranges, reinforcing the need for transparent reporting of deployability, subgroup behavior, calibration, and local threshold selection prior to routine adoption (13).

## ECG foundation models

ECG foundation models (FMs) are typically pretrained on large datasets of unlabeled or weakly labeled ECG waveforms using self-supervised or representation-learning objectives to capture generalizable electrophysiologic features. These pretrained representations can then be fine-tuned for downstream tasks (including LVSD screening) with substantially fewer echo-labeled examples than training task-specific models from scratch (7–10). Architectures and pretraining methods vary across FMs (e.g., convolutional neural network/RegNet- and transformer-based backbones), often incorporating ECG-specific augmentations such as lead masking, noise injection, or baseline-wander perturbations to improve robustness to real-world acquisition variability (7–10). Public resources such as MIMIC-IV-ECG or EchoNext with paired diagnostic 12-lead ECG recordings linked to clinical data are increasingly used for benchmarking and reproducible evaluation, although cross-study comparability still depends on cohort selection, label definitions, and ECG–echo timing (14–16).

ECGFounder is a large-scale, multi-institutional FM trained on >10 million ECGs across 150 label categories. External evaluations reported cross-domain generalization, and fine-tuned models performed competitively across multiple ECG tasks with transfer to LVEF classification/regression, illustrating how scale and heterogeneous real-world labels can yield useful ECG representations despite label noise (7).

ECG-FM is an open-weight FM pretrained on ∼1.5 million 12-lead ECGs. After fine-tuning, it reported AUROC 0.929 for LVEF ≤40% on an independent institutional dataset, with strong label efficiency and evidence of cross-dataset transfer, features that may be particularly relevant for centers with limited local ECG–echocardiography pairs (8).

A further demonstration of self-supervised pretraining benefits was provided by Moody et al., who developed a vision transformer foundation model pretrained on 800,035 unlabeled ECGs from the MIMIC-IV-ECG database and fine-tuned on smaller task-specific labeled cohorts. For LVEF <35% classification, the model achieved an AUROC of 0.955 overall and 0.949 on external validation, where self-supervised pretraining outperformed the supervised counterpart (AUROC 0.949 vs. 0.894). Self-supervised pretraining improved performance in 11 of 12 evaluated clinical prediction tasks compared with conventional *de novo* supervised training, a pattern consistent across five cross-modality databases comprising three external and two internal cohorts (9).

The most direct head-to-head comparison of supervised and self-supervised foundation model approaches for LVEF ≤40% classification was reported by Nolin-Lapalme et al., who evaluated two open-source ECG foundation models, DeepECG-SL and DeepECG-SSL, across 11 geographically diverse datasets including four public repositories and seven institutional cohorts. While both models achieved equivalent performance for ECG rhythm interpretation, DeepECG-SSL demonstrated higher discrimination for LVEF ≤40% classification (AUROC 0.926 vs. 0.917, Δ = 0.009, *p* < 0.001), with the advantage of self-supervised pretraining widening as training dataset size decreased. This finding has direct relevance for centers with limited local ECG–echocardiography pairs (10).

One earlier, image-based foundation model, HeartBEiT, was reported by Vaid et al., who rendered each ECG as an image and pretrained a vision transformer by masked image modeling rather than operating on the raw waveform (17). Applied to low ejection fraction, HeartBEiT outperformed conventional convolutional baselines, with the largest advantage when labeled training data were most limited, consistent with the label-efficiency pattern reported for the signal-based models. Because it operates on ECG images instead of the 10-s, 12-lead signal that anchors this review, it is discussed here as an image-based precedent and is not grouped with the signal-based foundation models.

For LVSD screening and triage workflows, the potential advantages of FMs include improved transportability across datasets relative to training from scratch, and preliminary robustness to missing leads and noise. Nonetheless, those claims require careful external validation, calibration reporting, and prospective testing when deployed outside development settings.

## Clinical implementation across care settings

**Figure 2:**
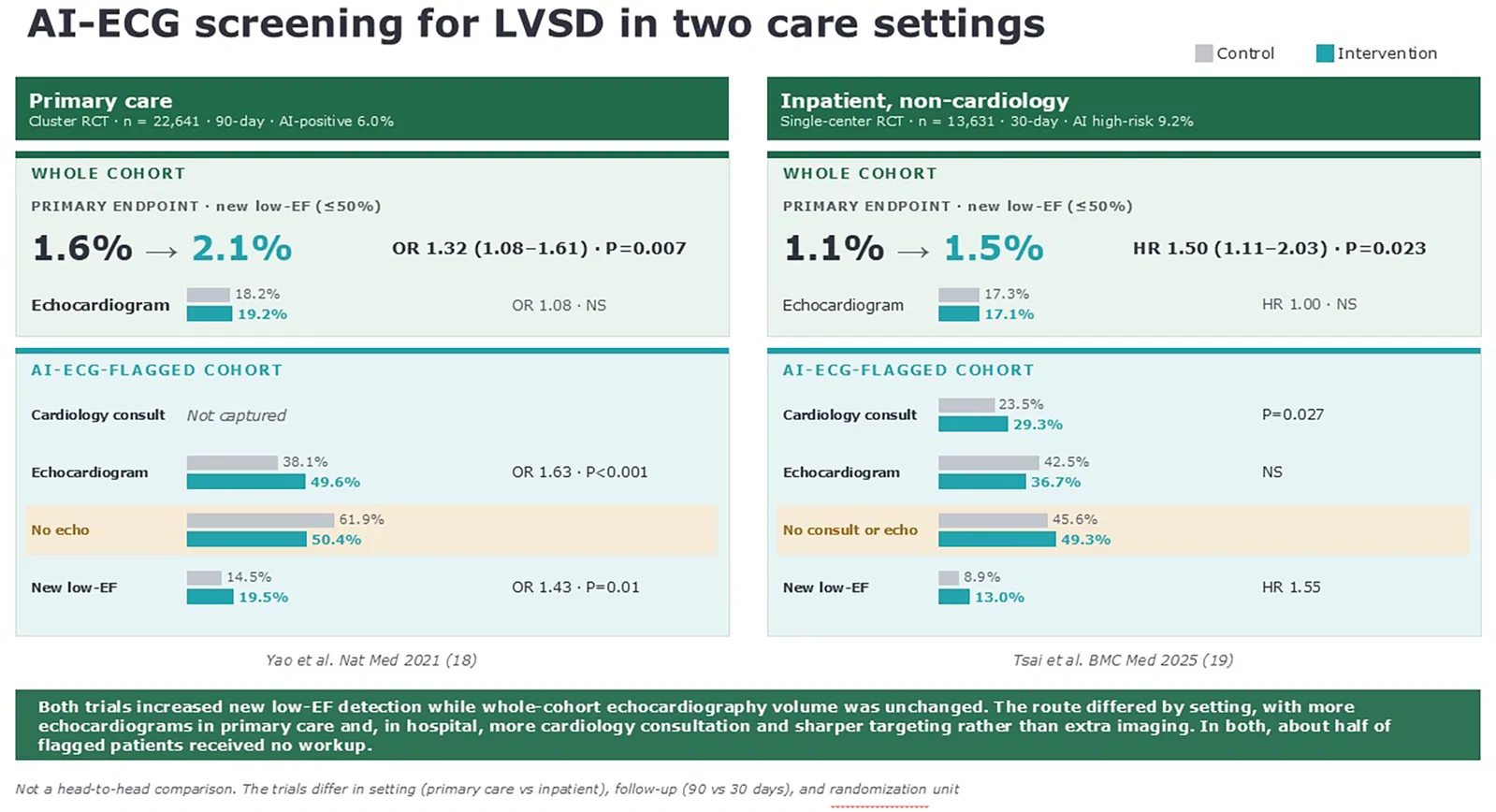
AI-ECG in two pragmatic randomized trials. In primary care (EAGLE; Yao et al.) and in a single-center non-cardiology inpatient setting (Tsai et al.), providing AI-ECG results increased new detection of low ejection fraction without raising whole-cohort echocardiography volume. The mechanism differed by setting, with more targeted echocardiography in primary care and more cardiology consultation in hospital, and in both trials roughly half of flagged patients received no workup. The two trials differ in setting, follow-up, and randomization unit and are not a head-to-head comparison. RCT, randomized controlled trial.

Two pragmatic RCTs demonstrate that AI-ECG can improve diagnostic efficiency when embedded in routine clinical workflows. In the EAGLE trial in primary care, providing AI-ECG results to clinicians increased identification of patients with low ejection fraction [≤50% capturing heart failure with mildly reduced and reduced ejection fraction (HFrEF)] and directed echocardiography preferentially to AI-positive patients, while overall echocardiography utilization was similar between groups (18). A subsequent inpatient RCT among non-cardiology wards replicated the diagnostic benefit but via a different mechanism. Rather than increasing echocardiography volume, AI alerts primarily raised cardiology consultation rates among high-risk patients, which improved the yield of echocardiography performed without increasing overall imaging utilization (19). Together, these trials suggest that the care pathway to diagnosis, that is, increased imaging in underdiagnosed outpatients versus more targeted specialist referral in closely monitored inpatients, depends on the clinical context (Figure 2). Of note, neither trial was designed to demonstrate downstream patient benefits. Prospective outcome-focused trials are needed to establish whether earlier AI-ECG–guided diagnosis translates into reduced HF hospitalizations, improved quality of life, and prolonged survival.

In the emergency department, an AI-ECG model applied to adults presenting with dyspnea demonstrated high negative predictive value (NPV) supporting a triage and prioritization role, outperforming NT-proBNP alone for detection of LVSD (20). Because predictive values are prevalence-dependent, a negative screen cannot exclude LVSD in higher-risk presentations. In practice, a high NPV may help de-prioritize immediate echocardiography in selected lower-risk patients when combined with symptoms, examination, and biomarkers, while modest positive predictive value (PPV) highlights the need for confirmatory imaging and careful resource allocation.

In pooled community cohorts, adding AI-ECG screens for systolic and diastolic dysfunction to PREVENT-HF improved near-term HF risk discrimination and reclassification across 1–10 years (4, 5). The retrospective, non-interventional design of these analyses, however, precludes conclusions about clinical outcome benefit. Establishing whether improved risk stratification translates into fewer HF hospitalizations or reduced mortality requires prospective, intervention-based trials (4).

Most prospective LVSD evaluations and clinical workflows have relied on 10-s 12-lead ECGs (7, 8, 10). Contemporary HF guidelines emphasize appropriate use of imaging and targeted testing, and AI-ECG should be implemented as clinician decision support with explicit operating thresholds, confirmatory pathways, and ongoing calibration and quality monitoring (21).

In the United States, FDA 510(k)–cleared machine learning–based notification software is available to support screening for reduced ejection fraction (LVEF ≤40%) from standard 12-lead ECGs, including the Anumana Low Ejection Fraction AI-ECG Algorithm (K232699, 2023) and Tempus ECG-Low EF (K250119, 2025) (22, 23). These tools provide clinician decision support rather than a definitive diagnosis, are not intended for patient monitoring, and should not be used on ECGs with paced rhythms. Additional labeling limitations (e.g., eligible populations and repeat-testing constraints) vary by product and should be followed (22, 23). A positive result may suggest the need for further clinical evaluation (e.g., echocardiography), while a negative result does not rule out low LVEF, particularly in higher-risk patients, and results should be interpreted alongside symptoms, history, and other tests.

## Practical challenges and open questions

Operating thresholds and calibration shape whether AI-ECG meaningfully helps clinicians, because AUROC alone does not indicate how the tool will perform at a chosen decision cut-point in a given population. Thresholds should be set to match the intended use (e.g., triage/prioritization vs. case-finding), checked against local prevalence and case-mix with recalibration when needed, and evaluated for added clinical value using measures such as reclassification reported in pooled-cohort HF-prediction studies (4–6). High NPV in emergency triage may help prioritize workflow in carefully selected lower-risk patients, but should not be used to defer evaluation when clinical suspicion is moderate/high or when other high-risk features are present (20).

Performance attenuation in the presence of atrial fibrillation (AF) and wide QRS complexes is a consistent finding across published AI-ECG models for LVSD detection. In an independent external validation at the Heart Center Leipzig, AUROC in the AF subgroup was 0.815 compared with 0.878 overall (24). A head-to-head comparison of four externally validated models similarly demonstrated reduced discrimination in AF and wide QRS ECGs across all models tested, though absolute performance remained good to excellent (13). The clinical significance of this limitation may be partly mitigated by the fact that both AF and bundle branch block are recognized indicators of potential structural heart disease that typically prompt echocardiographic evaluation regardless of an AI-ECG result. In this context, reduced model performance in these subgroups does not necessarily translate into a meaningful screening gap. Nevertheless, dedicated model development and validation in patients with AF (25) or wide QRS complex (26), or hybrid approaches integrating clinical data such as natriuretic peptides, age, and electrolytes alongside ECG waveform features (27) represent important directions for improving AI-ECG utility in those high-prevalence subgroups.

Explainability is increasingly recognized as a prerequisite for clinical adoption of AI-ECG, given the trade-off between model accuracy and interpretability that characterizes most deep learning approaches (28). Feature-attribution methods, including SHAP, integrated gradients, DeepLIFT, LIME, and Grad-CAM, have consistently localized AI-ECG decisions for reduced LVEF to QRS- and T-wave regions, with transformer-based architectures producing more focal saliency maps than convolutional networks for the same task (10, 17, 26). In a structured analysis using SHAP and dimensionality reduction, Katsushika and colleagues extracted six recognizable ECG criteria for reduced LVEF, including ventricular activation time prolongation in I/V5–6, low voltage, QTc prolongation, and pathological Q waves (28). Of note, cardiologists' ECG-reading accuracy improved from 62.9% to 73.9% after structured education on these criteria, supporting a learning loop between AI models and clinicians rather than a one-directional black-box prediction (28). In foundation models, self-supervised pretraining has been associated with more focal attention patterns than purely supervised training, although broader validation of clinical interpretability is required (10). At the same time, current saliency- and attribution-based explanations may be unreliable for individual predictions and have been shown to remain visually plausible even when applied to untrained networks or to inputs perturbed by adversarial attack, supporting their use for model development and audit rather than as a substitute for rigorous validation at the bedside (29). Direct mapping of learned features to specific pathophysiologic substrates such as fibrosis or remodeling remains inferential and warrants multimodal validation against echocardiographic, cardiac magnetic resonance imaging, and tissue-level correlates.

The AI-positive/echo-normal phenotype suggests detection of a pre-clinical electrical substrate (2, 24). Actionable protocols still require outcomes-focused trials to define when to order echocardiography, adjust therapy, or arrange follow-up (18, 19).

Although FM pretraining improves transfer, subgroup performance (age, sex, race/ethnicity, body mass index, rhythm, QRS width, renal function), device heterogeneity, and site-level calibration require explicit reporting at deployment and routine post-deployment monitoring, with local recalibration when needed (7, 8, 10). Large-scale datasets enable external testing and benchmarking, but broader international representation remains important. Echocardiographic EF measurements vary across vendors and observers. This heterogeneity can affect supervised training and model transportability, reinforcing the need for local calibration and clear EF threshold specification (≤35%, ≤40%, ≤50%) when reporting performance (3, 11, 30). Reported performance may also depend on the ECG–echocardiography matching window, which varies widely across studies from same-day pairing to intervals of 30–60 days. In a previous study, reported AUROC for low-EF detection rose with wider windows, although this trend was confounded by larger training-set size as the window widened (31). Beyond single-timepoint discrimination, short- and long-term reproducibility of FM-based LVSD predictions and their direct comparative performance against conventional supervised models using single ECGs per patient remain poorly characterized and represent priorities for retrospective and prospective evaluation.

Single-lead applications demonstrate encouraging early performance for incident HF risk (32). However, routine LVSD screening should continue to rely on validated 12-lead inputs until prospective, echocardiography-referenced studies confirm safety, efficacy, and cost-effectiveness for reduced-lead triage.

AI-ECG-based LVSD screening can generate false positives, leading to downstream testing, patient anxiety, and opportunity costs. Labels derived from echocardiography may be noisy because of measurement variability and temporal discordance between ECG and echo, affecting training and calibration. Performance can drift with distribution shift across devices, vendors, and acquisition settings. HF phenotype mix (HFrEF vs. heart failure with preserved ejection fraction) further warrants surveillance. Implementation should follow guidelines for judicious imaging, with clear action thresholds and post-deployment quality surveillance.

## Conclusions

AI-ECG offers a method to screen for LVSD across care settings. Newer foundation-model approaches may reduce labeled-data requirements and improve transportability, with early signals of robustness to noise or missing leads. Clinical use should rely on validated 12-lead ECG inputs, apply locally calibrated decision thresholds, and explicitly manage PPV/NPV trade-offs through confirmatory pathways rather than treating AI-ECG outputs as stand-alone diagnoses. Key next steps include outcome-focused trials, multimodal models that integrate ECG with clinical variables and natriuretic peptides, shared benchmarks with transparent reporting, and governance frameworks that support safe and equitable deployment. Prospective multi-site pragmatic trials should test whether AI-ECG–guided strategies accelerate diagnosis, reduce imaging resource consumption, increase appropriate initiation/intensification of guideline-directed therapy, and ultimately reduce HF hospitalization and all-cause mortality in a cost-effective manner. Additional priorities include prospective echocardiography-referenced validation of reduced-lead approaches, explicitly defined operating thresholds across PREVENT-HF risk tiers, routine subgroup calibration/fairness reporting, and post-deployment monitoring for drift and distribution shift.
